# Evaluation of the apical sealing ability of bioRoot RCS with Various Gutta-percha cones using spectrophotometry and scanning electron microscopy: an *in vitro* study

**DOI:** 10.3389/fdmed.2026.1888651

**Published:** 2026-08-12

**Authors:** Vinutha Manjunath, Natasha Noorani, Vidya G. Doddawad, SubbaRao V. Madhunapantula, Raghavendra Shanbhog, B. C Sulochanadevi

**Affiliations:** 1 Department of Conservative Dentistry and Endodontics, JSS Dental College and Hospital, Mysore, India; 2 Department of Biochemistry, JSS Medical College, Mysuru, India; 3 Department of Pediatric and Preventive Dentistry JSS Dental College and Hospital A Constituent College of JSS Academy of Higher Education and Research, Mysuru, India; 4 School of Public Health A Constituent College of JSS Academy of Higher Education and Research, Mysuru, India

**Keywords:** apical microleakage, bioceramic sealer, bioroot RCS, endodontic obturation, gutta-Percha, root canal sealing, scanning electron microscopy, spectrophotometry

## Abstract

**Background:**

The success of root canal therapy is highly dependent on achieving a well-sealed canal system to prevent apical microleakage and reinfection. The apical seal is primarily influenced by the sealing properties of the root canal sealer and the compatibility of the gutta-percha cones used for obturation.

**Objective:**

To evaluate the apical sealing ability of BioRoot RCS with various hydrophilic-coated gutta-percha cones using spectrophotometric analysis and to assess the obturation material–sealer–dentin interface using scanning electron microscopy (SEM).

**Materials and methods:**

Ninety-six extracted human premolars were randomly divided into four groups (*n* = 24) based on the type of gutta-percha cone. Twelve samples per group were subjected to dye extraction with 1% methylene blue, and leakage was measured using a spectrophotometer. The remaining samples were evaluated under a stereomicroscope and SEM to qualitatively assess the sealer–dentin interface. Statistical analysis was performed using one-way ANOVA and Tukey's *post hoc* test (*α* = 0.05).

**Results:**

The BioRoot RCS/bioceramic-coated gutta-percha group exhibited the lowest mean dye absorbance (0.0013 ± 0.0005), whereas the BioRoot RCS/gutta-percha group showed the highest (0.0057 ± 0.0010). SEM analysis demonstrated the smallest mean interfacial gap in the BioRoot RCS/bioceramic-coated gutta-percha group (2.76 ± 0.15 µm) and the largest in the BioRoot RCS/gutta-percha group (5.43 ± 0.16 µm). The differences among the groups were statistically significant (*p* < 0.05).

**Conclusion:**

Bioceramic-coated gutta-percha with BioRoot RCS provided superior sealing ability, confirmed by both spectrophotometric and SEM evaluations.

## Introduction

The pertinent objective of root canal treatment is to eliminate microbial infection and prevent future reinfection of the root canal system. To obtain this, a three-dimensional filling of the canal is required (1). The solid core material acts in synergism with the sealer to create a fluid-tight seal, leading to decreased risk of micro leakage (1, 2). Till date, gutta-percha (GP) is the most commonly used material. However, it is hydrophobic in nature and does not adhere to dentin. Therefore, to ensure a tight seal of the root filling, a sealer is required to help fill voids and flow into anatomical irregularities. Even so, sealers are subject to shrinkage, degrade over time, and do not bond to dentin. Consequently, the use of “a large amount of core material with the thinnest layer of sealer” is recommended to improve the quality of the filling (3).

Recently, “hydrophilic root canal obturating systems” have been introduced (4). They consist of hydrophilic sealers that use the moisture in the dentinal tubules to initiate and complete the setting reaction, as well as hydrophilic substance-coated core materials, which reduce the number of interfaces and improve bonding (4). These sealers bond to the dentinal walls as well as the hydrophilic core materials, thus creating a monobloc, thereby providing a gap-free single-cone sealer-based obturation technique with better seal and reduced microleakage (5).

The methacrylate resin-based sealers, such as Epiphany and EndoREZ, were among the earliest sealers to be introduced. The EndoREZ (ER) obturating system (Ultradent Products Inc, South Jordan, UT., USA), consisting of EndoREZ sealer, also claims to bond chemically when used with EndoREZ points (resin-coated Gutta Percha), establishing a continuous adhesion between all the materials (6). Later, “ActiV GP Precision” Obturating System (“Brasseler USA, Savannah, GA”) was introduced, which consists of an ActiV GP point coated with 2 µm of Glass Ionomer(GI) particles on its surface and GI-based sealer (7)*.* Recently, Bioceramic obturating systems have gained popularity. Notably among them is the BioRoot™ RCS (Septodont), which is a bioactive mineral root canal sealer (RCS) with no customised root canal GP, and the Endosequence (Brasseler USA, Savannah, GA), which consists of bio ceramic coated GP points and bio ceramic (BC) sealer (3).

Among the available bioceramic sealers, BioRoot RCS (Septodont, France) was selected for the present study because of its bioactive calcium silicate composition and its documented ability to release calcium ions, induce mineralization, and form a chemical bond with dentin through the deposition of hydroxyapatite. In addition, BioRoot RCS has demonstrated favorable sealing ability, biocompatibility, and bioactivity in previous studies. Although iRoot SP is a widely used premixed bioceramic sealer, BioRoot RCS was chosen because its bioactive properties and interaction with hydrophilic obturation systems make it particularly suitable for evaluating the influence of different coated gutta-percha cones on apical sealing ability.

Several studies have been done to evaluate and compare the apical microleakage of conventional sealers with different coated gutta-percha points and bioceramic sealers with conventional gutta-percha points. But this study was an attempt to evaluate microleakage of different coated hydrophilic gutta-percha points with hydrophilic bioceramic sealers. Therefore, the purpose of this study was to evaluate the apical sealing ability of BioRoot RCS/ BC coated GP points, BioRoot RCS/ ActiV GP, BioRoot RCS/EndoREZ points and BioRoot RCS/ gutta-percha obturation by assessing the microleakage using a Spectrophotometer; to evaluate the apical extent of the microleakage using the Stereomicroscope and to evaluate the obturation material-sealer-dentin interface using Scanning Electron Microscope.

## Materials and methods

The present study was an *in-vitro* comparative study, which was conducted in the Department of Conservative Dentistry and Endodontics. All procedures were performed after the ethical clearance was obtained from the Institutional Ethics Committee (JSSDCH IEC/23/2018). 96 extracted noncarious premolar teeth were used. It was carried out for 12 months from June 2019 to May 2020.

### Sample calculation

The sample size consisted of 12 (*n* = 12) and was calculated by the mean comparison formula, considering an *α* = 0.05 and a statistical power of 1−*β* = 0.80. In addition, the study units were selected by simple random sampling by computer-generated randomization, without replacement. The formula used for the sample size is shown below.

$$n=\frac{2{\left(Z_{\alpha /2}+Z_{\beta }\right)}^{2}\;\sigma^{2}}{\Delta^{2}}$$

All samples were blinded and randomly divided into four experimental groups (*n* = 24) according to the restorative materials used. Each group consisted of 12 teeth for methylene blue dye application to measure microleakage using spectrophotometry, and 12 teeth for assessing the sealer–dentin interface using scanning electron microscopy (SEM).

A total of 96 (*N* = 96) extracted single-rooted human upper and lower premolars for orthodontic purposes were obtained for the present study after obtaining the patient’s consent.

Only single-rooted premolars with a single canal, fully developed apices, and no evidence of root caries, cracks, fractures, internal or external resorption, calcification, or previous endodontic treatment were included.

### Preparation of the specimen

Disinfection of the teeth was done using 2.5% sodium hypochlorite solution for 1 h, post which they were stored in normal saline. Soft tissue residues and calculi were removed using an ultrasonic scaler and were used within a month of extraction. The cusps of the teeth were flattened using a diamond disc. The access opening was done. Patency was evaluated using a #10 K file. The actual length of each tooth was determined, and the working length was established by subtracting 0.5 mm from it. Canals were prepared using #10, #15, and #20 K files, respectively, followed by Protaper Gold up to F4 using the crown-down technique.

Following biomechanical preparation to the ProTaper Universal F4 finishing file, the corresponding master cone for each experimental group was selected according to the manufacturer's recommendations. The master cone was adjusted to the predetermined working length, and apical fit was confirmed by a slight tug-back at that length before obturation. Only specimens demonstrating satisfactory apical adaptation were included for further evaluation.

During instrumentation, 17% ethylenediaminetetraacetic (EDTA) gel was used as a lubricant, and the canals were irrigated using 5 mL of 5.25% Sodium Hypochlorite (*N*aOCl) using a 27-gauge needle. After complete instrumentation, each canal was irrigated using passive ultrasonic/ manual agitation with 1 mL 17% EDTA for one minute, followed by saline and then with 2 mL of 5.25% NaOCl solution for 30s with a final rinse of 5 mL distilled water to remove any traces of NaOCl. Paper points were used to dry the canals. The teeth were then randomly categorized into four groups with 24 samples per group, depending on the core material used for obturation. Teeth were obturated using BioRoot RCS sealer using a cold hydraulic single cone obturation technique.

The four groups were as follows:

Group 1 (*n* = 24): BioRoot RCS/ EndoSequence BC Points; (35/0.6)
Group 2 (*n* = 24): BioRoot RCS/ ActiV GP Points; (35/.06)
Group 3 (*n* = 24): BioRoot RCS/ EndoREZ Points (35/.06)
Group 4 (*n* = 24): BioRoot RCS/ Gutta Percha. (35/.06)

All the restored teeth were subjected to thermocycling for “500 cycles in a water bath at 5 °C ± 2 °C (cold cycle) and 55 °C ± 2 °C (hot cycle) with a dwell time of 30 s.

BioRoot RCS sealer (Septodont, Saint-Maur-des-Fossés, France) was used in combination with EndoSequence BC Points (Brasseler USA, Savannah, GA, USA), ActiV GP Points (Brasseler USA, Savannah, GA, USA), EndoREZ Points (Ultradent Products Inc., South Jordan, UT, USA), and conventional gutta-percha cones (Dentsply Sirona, North Carolina). The manufacturer details and corresponding batch/lot numbers of all materials used in the study were recorded to ensure standardization and reproducibility.

### Microleakage assessment by spectrophotometric analysis using the dye extraction method

**Figure 1:**
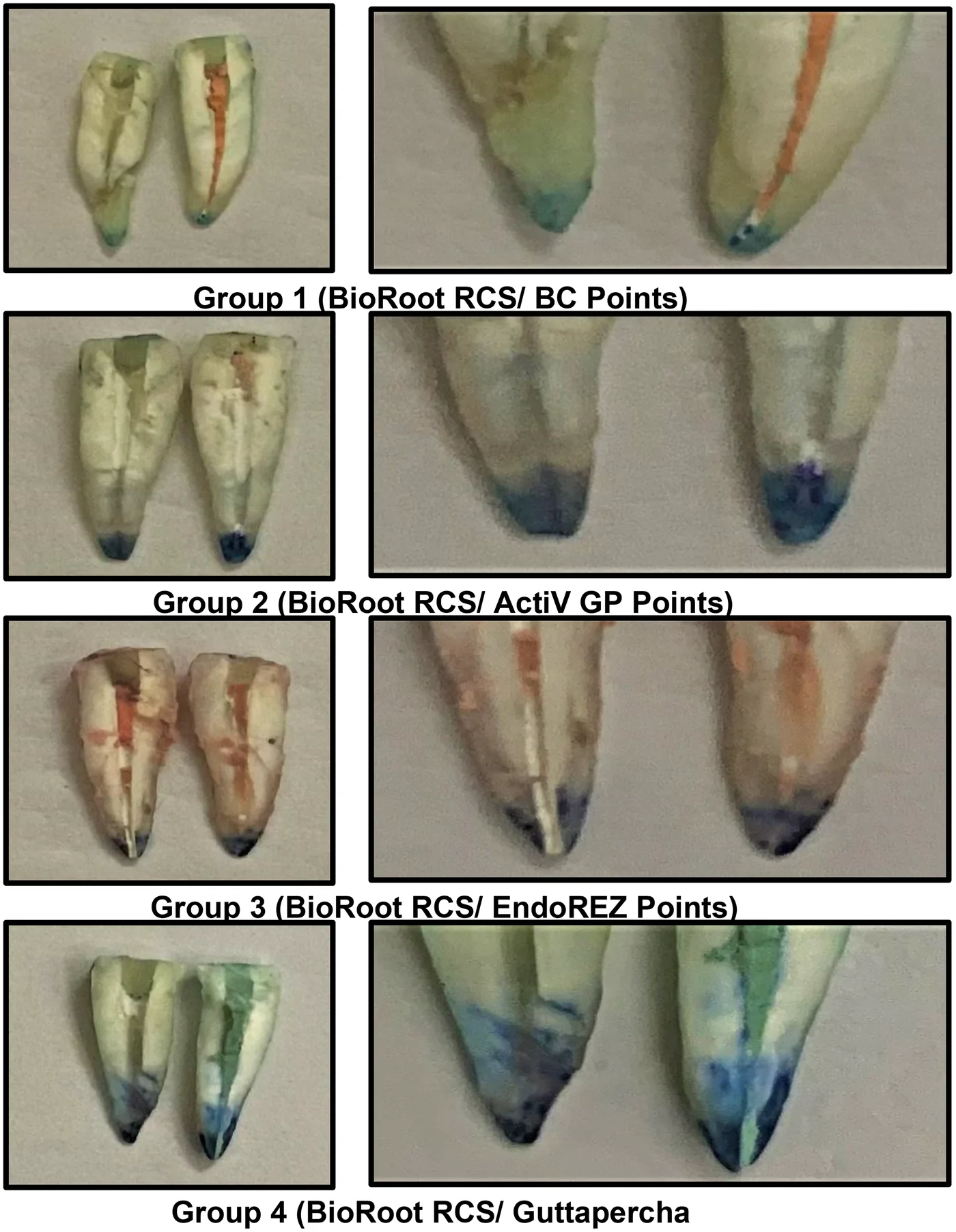
Macroscopic Spectrophotometer images of the apical third of the root.

12 samples per group were used for this test. The dye extraction method employs a spectrophotometer to assess the absorbance of leaked dye. The teeth were coated with two layers of nail varnish, leaving a 2 mm area of the apical portion exposed. The samples were immersed for one day at 37 °C in a neutral buffer solution containing 1% methylene blue. After removal from the dye solution, all specimens were rinsed thoroughly under running water for 30 min. The nail varnish was completely removed using a BP blade and polishing discs, and the external root surfaces were cleaned with Sof-Lex discs to eliminate any residual superficial dye before immersion in nitric acid. These procedures were performed to minimize external dye contamination. The samples were then placed in a sterile container with 6 mL of 65% nitric acid for 3 days, followed by centrifugation of this solution at 5,000 rpm for 15 min to separate debris from the extracted dye. All procedures were conducted in a light-proof environment to prevent photo-bleaching. The supernatant was transferred to a cuvette of the Eppendorf spectrophotometer (BioSpectrometer, Germany) using micro pipettes, and the absorbance of each sample was measured at 670 nm wavelength. Before this, the absorbance values of known concentrations of methylene blue dye were determined. Standards with concentrations of 0%, 0.005%, 0.01%, and 0.02%—near the expected concentration in the unknown samples—were prepared (Figure 1). Each standard was analyzed with the spectrophotometer, producing a series of absorbance readings. From these data, a calibration curve was plotted to determine the unknown concentration relative to the standards. The correlation coefficient (R) between dye concentration and absorbance of the standard solutions was calculated to estimate the dye concentration in the experimental samples. A linear regression was established and expressed as:

y=48.354x–0.0316

where y is the absorbance and x is the dye concentration.

The calibration curve was constructed using methylene blue concentrations ranging from 0% to 0.02%. Linear regression demonstrated excellent linearity (R^2^ = 0.998), and all specimen absorbance values fell within the linear calibration range.

### Obturating material-sealer-dentin interface assessment using scanning electron microscope

**Figure 2:**
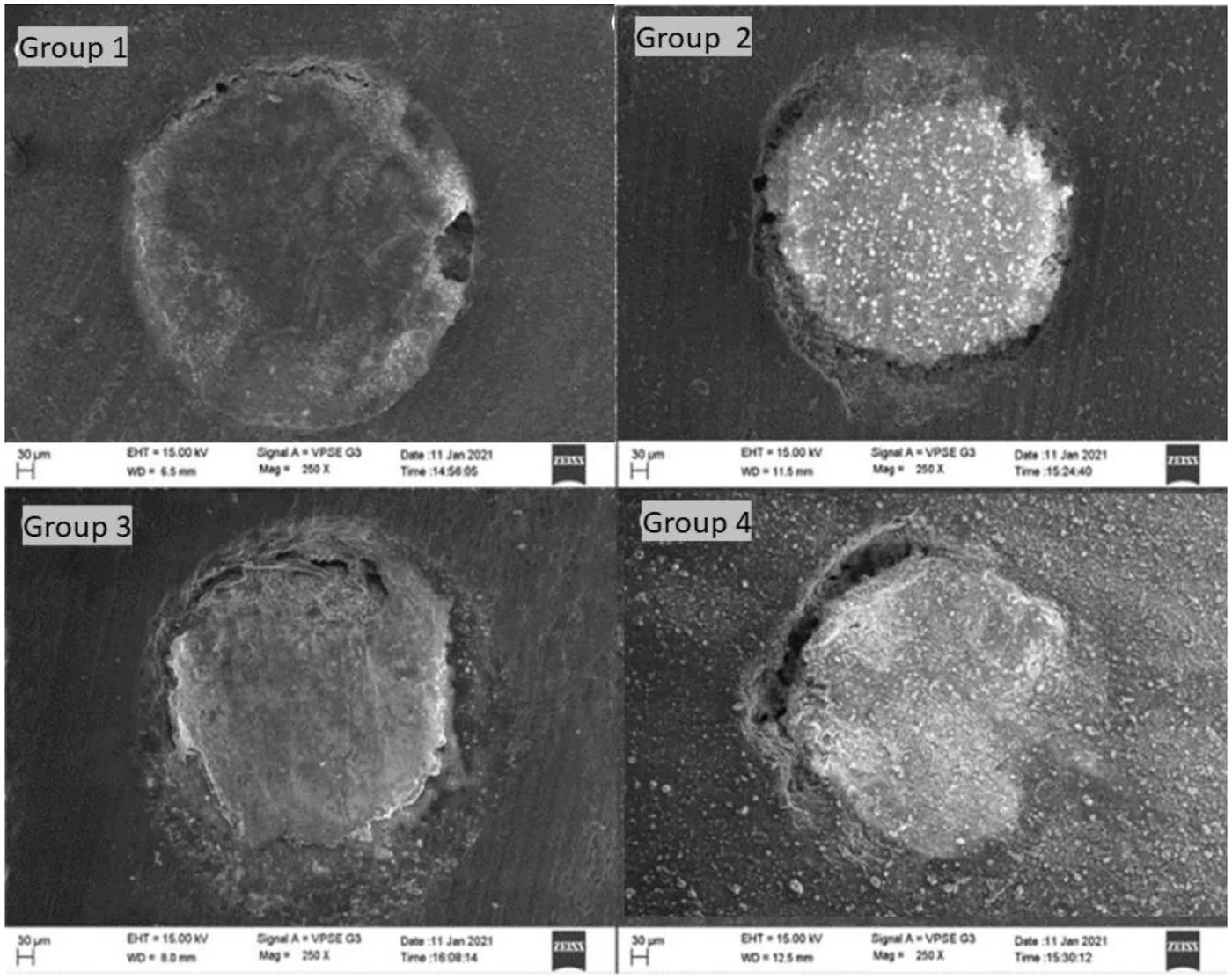
Representative scanning electron microscopy (SEM) images of the root dentin–sealer–core material interface for the four experimental groups (×1,000 magnification).

An independent investigator assigned all specimens coded identifiers. The examiner responsible for SEM image acquisition and gap measurements was blinded to the experimental group allocation during evaluation. Roots were transversely sectioned using a slow-speed, water-cooled mounted saw 2–3 mm above the apex. The samples were mounted on aluminum stubs and coated with carbon for insulation. The specimens were examined using a scanning electron microscope (ZEISS EVO LS 15, Germany). SEM images were obtained at ×1,000 magnification. Three representative images were captured from each specimen at the sealer–dentin interface. Gap measurements were performed using the image analysis software integrated with the SEM system (Image J). Three measurements were recorded from each image, and the values were expressed in micrometres (µm). The mean value of all measurements for each specimen was used in the statistical analysis. The interfacial gap interval was defined as the perpendicular distance between the root dentin and the obturation material/sealer interface. A smaller gap interval indicated better interfacial adaptation and sealing ability, whereas a larger gap interval indicated poorer adaptation (Figure 2).

**Figure 3:**
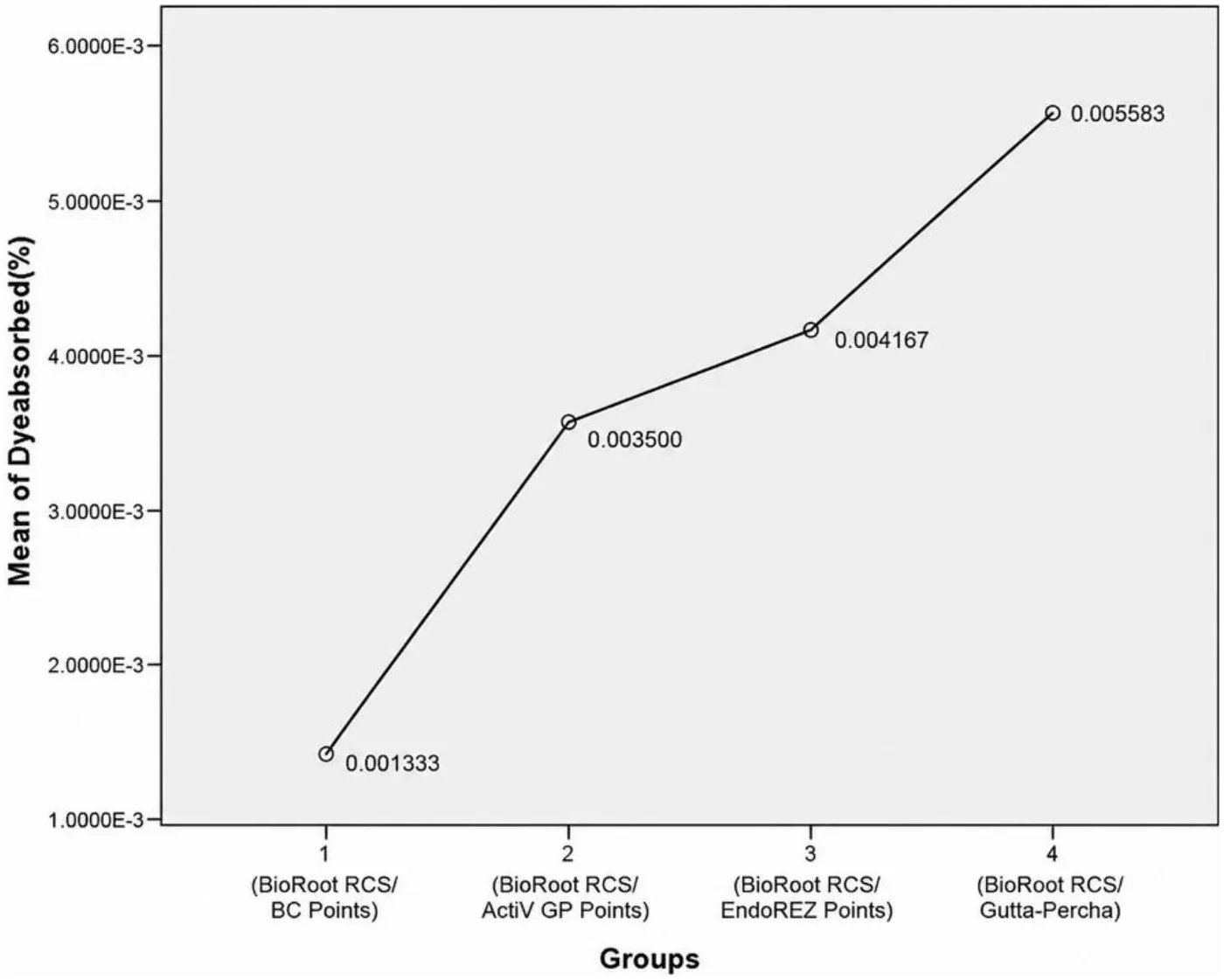
Comparison of the mean concentration of methylene blue dye absorbed at the root dentin–sealer interface among the four experimental groups.

**Figure 4:**
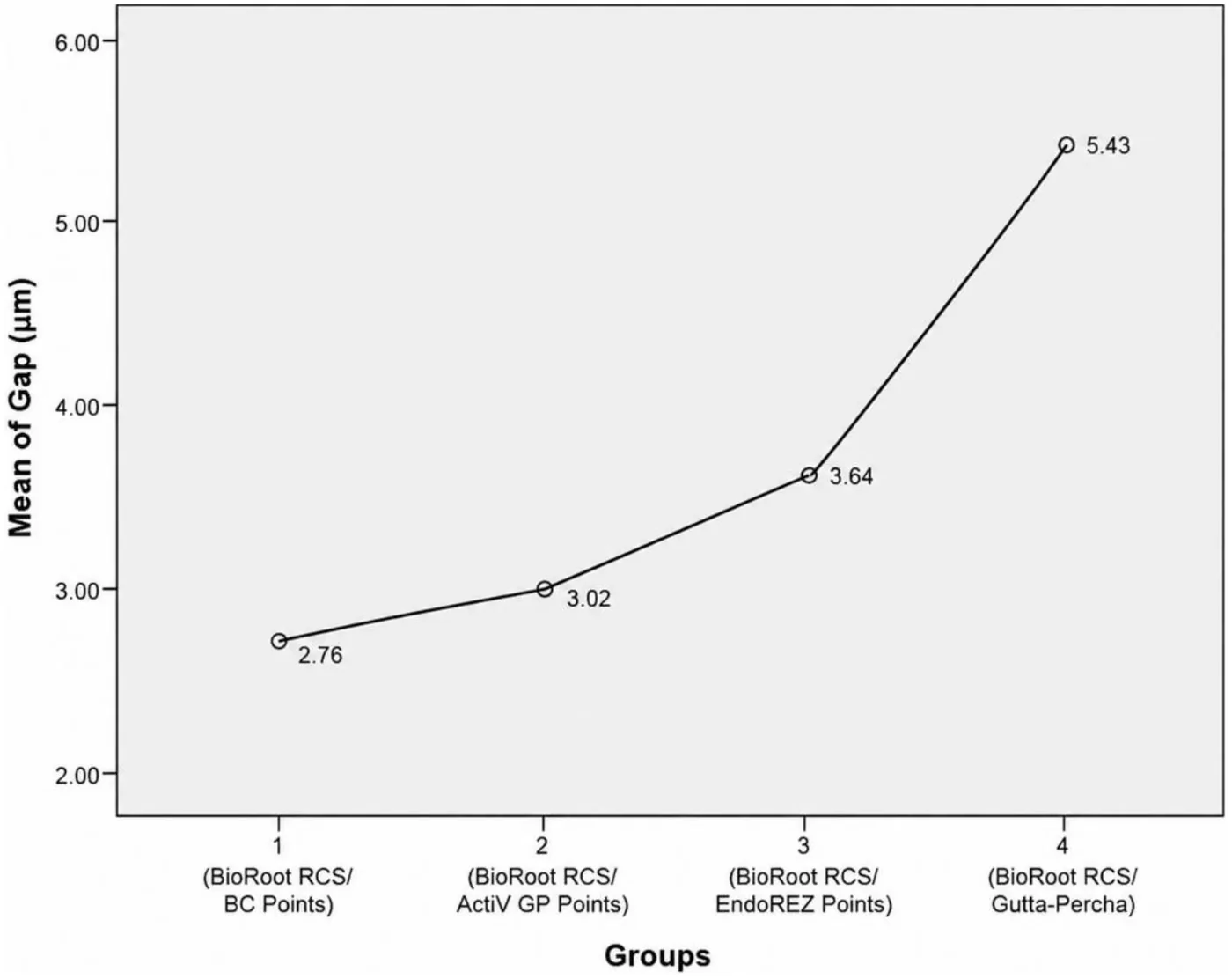
Comparison of the mean interfacial gap at the root dentin–sealer interface among the four experimental groups.

The SEM was calibrated using the manufacturer's built-in calibration scale prior to image acquisition. All measurements were performed using the calibrated scale bar under identical imaging parameters. Gap measurements were expressed in micrometres (µm). All measurements were performed by a single blinded examiner. Therefore, intra-examiner and inter-examiner reliability were not assessed, which is acknowledged as a limitation of the present study.

### Statistical analysis

The mean of the concentration of dye absorbed in percentage (%) was calculated for each group, and the mean % was taken as one unit for statistical analysis. Statistical analysis was done using the SPSS Program version 22.0 (Statistical Package for the Social Sciences; SPSS, Chicago, IL, USA). Data was analysed using one-way analysis of variance (ANOVA). For multiple comparisons, *post-hoc* Tukey's test was performed. The level of significance was fixed at *p* ≤ 0.05.

## Results

 Table 1 compares dye absorption at the root dentin-sealer interface for four obturation groups. Group 1 (BioRoot RCS/BC Points) shows the lowest mean absorption (0.001333) with minimal variability, indicating superior sealing. Group 4 (BioRoot RCS/gutta-percha) exhibits the highest absorption (0.005583), suggesting the least effective seal among the groups (Figure 3). Table 2 shows significant differences in dye absorption between the groups (F = 45.610, *p* < 0.001). Table 3 reveals significant differences in dye absorption between Group 1 and Groups 2, 3, and 4, where the *p* value is <0.001. Group 2 differs significantly from Group 4, while Group 3 differs significantly from Group 4 (*p* < 0.001)but not from Group 2 (*p* = 0.287). Table 4 presents the mean ± SD values of the interfacial gap at the root dentin–sealer interface for the four obturation groups. Group 1 (BioRoot RCS/BC Points) exhibited the largest mean gap interval (5.43 ± 0.16 µm), followed by Group 2 (BioRoot RCS/ActiV GP Points) (3.64 ± 0.16 µm), Group 3 (BioRoot RCS/EndoREZ Points) (3.02 ± 0.19 µm), and Group 4 (BioRoot RCS/Gutta-Percha) (2.76 ± 0.15 µm), which demonstrated the smallest mean gap interval (Figure 4). Table 5 presents the one-way ANOVA revealed a statistically significant difference among the four groups (F = 93.07, *p* < 0.001). Tukey's *post hoc* analysis showed significant differences between Group 1 and Groups 2, 3, and 4 (*p* < 0.001), between Group 2 and Group 4 (*p* < 0.001), and between Group 3 and Group 4 (*p* < 0.001), whereas no statistically significant difference was observed between Groups 2 and 3 (*p* = 0.402) as shown in the Table 6.

**Table 1:** Mean ± SD values of the concentration of methylene blue dye absorbed at root dentin and sealer interface following obturation with four group.

Group	N	Mean	Std. Deviation
Group 1 (BioRoot RCS/ BC Points)	12	0.001333	0.0004924
Group 2 (BioRoot RCS/ ActiV GP points)	12	0.003500	0.0007977
Group 3 (BioRoot RCS/ EndoREZ points)	12	0.004167	0.0009374
Group 4 (BioRoot RCS/ Gutta-Percha)	12	0.005583	0.0012401

**Table 2:** Intergroup comparison of concentration of methylene blue absorbed at root dentin and sealer interface following obturation with four group using one-way ANOVA.

Source of variation	Sum of Squares	df	Mean Square	F	Sig.
Between Groups	0.000	3	.000	45.610	.000
Within Groups	0.000	44	.000		
Total	0.000	47			

**Table 3:** Multiple comparisons of concentration of methylene blue dye absorbed in % using *post hoc* tukey's test.

Dependent Variable: Conc. Of Methylene blue absorbed in %				
Tukey HSD				
(I) Group Code	(J) Group Code	Mean Difference (I-J)	p	Sig.
1	2	−0.0021667^a^	.000	Significant
	3	−0.0028333^a^	.000	Significant
	4	−0.0042500^a^	.000	Significant
2	3	−0.0006667	.287	Not Significant
	4	−0.0020833^a^	.000	Significant
3	4	−0.0014167^a^	.002	Significant

a The mean difference is significant at the 0.05 level.

**Table 4:** Mean ± SD values of the gap interval at root dentin and sealer interface following obturation with four group.

Group	N	Mean	Std. Deviation
Group 1 (BioRoot RCS/ BC Points)	12	5.43	0.16
Group 2 (BioRoot RCS/ ActiV GP points)	12	3.64	0.16
Group 3 (BioRoot RCS/ EndoREZ points)	12	3.02	0.19
Group 4 (BioRoot RCS/ Gutta-Percha)	12	2.76	0.15

**Table 5:** Intergroup comparison of gap interval at root dentin and sealer interface following obturation with four group using one-way ANOVA.

Source of variation	Sum of Squares	df	Mean Square	F	Sig.
Between Groups	58.912	3	19.637	93.07	<0.001
Within Groups	9.292	44	0.211		
Total	68.203	47			

**Table 6:** Multiple comparisons of gap interval at root dentin and sealer interface following obturation using *post hoc* tukey's test.

Dependent Variable: Gap interval at root dentin and sealer				
Tukey HSD				
(I) Group Code	(J) Group Code	Mean Difference (I-J)	P	Sig.
1	2	1.79	*<0.001	Significant
	3	2.41	*<0.001	Significant
	4	2.67	*<0.001	Significant
2	3	0.62	0.402	Not Significant
	4	0.88	*<0.001	Significant
3	4	0.26	*<0.001	Significant

* The mean difference is significant at the 0.05 level.

A total of 48 specimens (12 per group) were included for each quantitative outcome measure. One-way ANOVA was performed with four independent groups (df between=3, df within=44, total df = 47), followed by Tukey's *post hoc* test for pairwise comparisons. Statistical significance was set at *p* ≤ 0.05.

## Discussion

The terminal goal of obturation is “to utilize materials and techniques capable of densely filling the entire root canal to achieve an impermeable fluid-tight seal from the apical segment to the canal orifice to stave off re-infection of root canals that have been biomechanically cleaned and shaped.” (5). Apical leakage is known to be the main reason for root canal failure. Regardless of the obturation technique, the main function of the sealers and the core material is to provide a long-lasting seal along the canal wall. Hence, the root canal filling should seal the canal space apically to prevent microleakage (8). The major disadvantage of GP is its inability to reinforce endodontically treated teeth as it does not adhere to the root dentin and is incapable of forming a monobloc. GP is not capable of forming a monobloc even when used with a resin-based sealer, such as AH plus, as there is no bond between the sealer and GP. Several studies done *in vitro* have shown that teeth obturated with GP have high leakage values. Efforts have been made to introduce newer hydrophilic obturation materials (sealer and GP) that adhere to each other as well as to dentin to provide a superior seal, thus forming a monobloc. Therefore, the present study was designed to evaluate the microleakage of various experimental groups using the dye extraction method followed by spectrophotometric analysis. Additionally, SEM was utilized to assess the obturating material–sealer–dentin interface and to correlate interfacial gap measurements with the corresponding microleakage values.

The present study used methylene blue for the assessment of microleakage in all four experimental groups. The dye extraction method used involved staining the sample with methylene blue, followed by dissolving of sample in nitric acid (9). All the dye that leaked through the apex was recovered by dissolving in acid, the optical density of which was measured using a spectrophotometer. Also, quantitative measures were obtained, thus providing reliable results in microleakage studies (10). It is preferred as it has a molecular weight (318.85) lower than that of bacterial endotoxins and a particle size of 0.1–2 µm, which is smaller/ comparable to the size range of various endodontic microbes. This makes it an indicator for the passage of microorganisms and their toxins. This dye is also widely used due to its low cost and high degree of staining. However, at times, it is difficult to observe the maximum penetration points. A minimum of 24 h of immersion of the samples was done in this study to ensure the penetration of the dye apically into the interface of the tooth-gutta-percha-sealer (11). In the dye extraction method, the teeth were dipped in nitric acid for 3 days, as per the protocol followed by previous researchers, as the complete dissolution of dye into the acid takes 3 days (12). Before staining the sample, varnish was applied on the surface 2 mm short of the apex to allow the dye to penetrate the lateral and accessory canals to assess leakage, as 73.5% of ramifications are found in the apical 1–2 mm of the canal (13).

The present study compared the apical microleakage of a newly introduced hydrophilic bioceramic root canal sealer, BioRoot RCS, with different hydrophilic coated gutta-percha points like EndoSequence BC, ActiV GP and EndoREZ points. The findings of the present study indicated that all the combinations of sealer and core material leaked, thus showing that no complete seal occurred in any of the systems. However, the bioceramic sealer/ bioceramic EndoSequence BC group showed the lowest concentration of dye penetration, while the bioceramic sealer with gutta-percha showed the highest value. No significant difference in microleakage was seen between Bioceramic sealer/ ActiV GP point and Bioceramic sealer/ EndoREZ groups. Microleakage was assessed using spectrophotometric analysis with the dye extraction method, while SEM was employed to evaluate the core–sealer–dentin interface (14, 15). Both methods yielded similar results.

BioRoot RCS used in this study is a hydrophilic calcium silicate bioceramic sealer with multiple unique properties and is a material of choice over other sealers. It has a smaller particle size and high flowability with a low contact angle that enables it to spread over the dentinal walls and penetrate the tubules. It utilizes the humidity in the canal and tubules, thereby initiating its setting by the production of hydroxyapatite, forming a bond with root dentin. It chemically bonds to dentine by alkaline etching (due to the alkalinity of the sealer), and a mineral infiltration zone is formed at the interface of the dentine in contact with the material (16).

Its expansion of 0.20% allows the formation of a gap-free obturation (17), (18) stated that bioceramic root canal filling materials can expand by 0.2%–6% of the initial volume. Water sorption induces some expansion and makes a strong contribution to the sealing capacity (18). Chisnoiu et al. stated that these sealers could penetrate tubules and polymerize, leading to the formation of a hybrid layer. This formation was encouraged by the presence of hydrophilic monomers and solvents present in bioceramics. It is an apt material to be used in conjunction with the “single cone technique,” where the gutta-percha cone acts as a carrier of the sealer into the canal. Hence, a single cone cold hydraulic condensation technique was used in this study (19).

Despite all these properties, all the groups obturated using this sealer showed leakage. The sealer, being hydrophilic in nature, is advocated to be used with hydrophilic bioceramic-coated gutta-percha points for better results. However, the specimens obturated using BioRoot RCS/EndoSequence BC also showed leakage, though the values were the lowest. This could be attributed to the irrigation protocol used. EDTA 17% was used in this study for the removal of the smear layer. It is known to decrease the wettability of dentinal walls, giving rise to an environment that aids the adhesion of hydrophobic materials but prohibits the adhesion of hydrophilic materials (20). EDTA, being an established calcium chelator, affects the chemistry of calcium-containing bioceramic sealers by reducing the interaction of calcium ions with dentine and the deposit of *β*-calcium phosphate in the sealers. Therefore, the choice of irrigation protocol is important when using bioceramic sealers (16). In the current study, although distilled water was used as the final rinse, it might have been insufficient to flush away EDTA and increase the wetting ability of dentin.

Another reason leading to leakage in all groups could be that BioRoot RCS is not a premixed sealer and requires to be mixed manually by hand. This could have led to the discrepancy and error in the consistency of the final mix, leading to microleakage.

Group 1 showed the lowest microleakage mean value compared to the other groups. According to the manufacturer, bioceramic sealers are advocated to be used with bioceramic-coated gutta-percha for better results. This finding can be attributed to the chemical bonding and mechanical impregnation of a uniform coating of bioceramic nanoparticles over the surface of gutta-percha, which, being similar to the chemistry and surface energy of the hydrophilic sealer, enhances the sealing ability and forms a tertiary monobloc (15). Also, the high flowability of the sealer and the hydraulic pressure resulting from the insertion of the hydrophilic coated gutta-percha into the canal create an intimate contact between the sealer and dentin, allowing the formation of an interfacial hydroxyapatite layer, thus creating a chemical bond (8).

Group 4 showed the highest microleakage values, which, though clinically not truly relevant, were statistically significant and higher as compared to Group 1. In this group, gutta-percha was used as the core material. It is hydrophobic and does not bond to dentin. Also, the sealer being hydrophilic could bond to dentin but failed to adhere to gutta-percha, leading to the formation of gaps and hence microleakage. This was in accordance with a study conducted by Abdel et al, who stated that the changing of dentin surface energy due to EDTA solution significantly reduced its wetting ability thereby reducing the adhesion of hydrophilic root canal sealer, especially when used with a core material which is hydrophobic and has a dimensional stability such as gutta-percha. This could explain the high leakage mean value observed in the canals obturated with single-cone gutta-percha/BioRoot RCS (8).

Specimens obturated with ActiV GP points and EndoREZ points did not show any significant difference among themselves and resulted in higher leakage values than group 1, but lower as compared to group 4. ActiV GP points are hydrophilic and are known to form a tertiary monobloc system when used with glass ionomer ActiV GP sealer. “It consists of a conventional gutta-percha cone surface coated with glass-ionomer. Bonding to root dentin is provided by the ability of glass ionomer sealers to adhere to the hydroxyapatite component of dentin. This system is recommended to be used with a single-cone obturation technique.” (21). Cimpean et al. advocated its use with bioceramic sealer as well, stating that bonding will occur between the bioceramic sealer and the ceramic particles in the ActiV GP cones, thus achieving a true bond between the root canal wall and the master cone (22). However, it has been reported that the coating of GI filler on the surface of ActiV GP is nonhomogeneous, which may contribute to less favorable bonding, thus leading to increased microleakage (7).

EndoREZ points are resin-coated gutta-percha points that claim to produce a monobloc when used with methacrylate resin-based sealers. According to Darrag et al, the chemical bond between the polyisoprene of gutta-percha and the polybutadiene of resin coating appeared to be stronger than the bond between the methacrylate component of the coating and the sealer. This could be attributed to the removal of the essential oxygen inhibition layer formed during the coating to avoid sticking the cones during storage. It was also observed that the sealer showed polymerization shrinkage (23). These points showing leakage could be attributed to the fact that, although hydrophilic, they do not have a similar chemistry and surface energy of their resin coating to that of the bioceramic sealer, which prevents the formation of a chemical bond. Hence, the manufacturer has instructed it to be used with methacrylate resin-based sealers only. These points, when used with their standard resin sealer, also depicted leakage, lack of bond, and gap formation due to polymerization shrinkage, which explains why it showed higher leakage when used with a non-recommended bioceramic sealer (24).

The dye extraction technique employed in the present study has been widely used in endodontic microleakage research because it provides quantitative assessment of dye penetration. Nevertheless, since the method involves nitric acid digestion of the specimens, dissolution of calcium silicate-based sealers or coated gutta-percha materials cannot be completely excluded. Although the external root surfaces were thoroughly cleaned before acid digestion to minimize superficial dye contamination, a minor material-dependent influence on dye recovery remains possible. Therefore, the findings should be interpreted in conjunction with the SEM observations, which demonstrated similar trends in interfacial adaptation.

The differences in SEM surface morphology among the experimental groups can be explained by the inherent characteristics of the obturation materials. EndoSequence BC Points possess a relatively uniform bioceramic coating that provides a homogeneous interface with BioRoot RCS. EndoREZ Points have a resin-coated surface, resulting in a comparatively smoother appearance. In contrast, ActiV GP Points are coated with glass-ionomer particles that have been reported to produce a non-uniform surface, while conventional gutta-percha lacks any surface coating. These material-dependent surface characteristics account for the differences observed in the SEM micrographs and may influence the adaptation at the sealer–dentin interface.

### Limitations of the study

This *in-vitro* study has limitations, including the absence of clinical conditions such as occlusal forces and saliva. Although thermocycling was performed to simulate thermal stresses, long-term aging and mechanical loading were not evaluated, which may limit extrapolation of the findings to long-term clinical conditions. Each examiner's calibration and reliability testing was not performed. The use of extracted teeth and a single sealer limits generalizability. Although standardized selection criteria and randomization were employed, minor variations in root anatomy, canal morphology, and tooth dimensions among extracted human premolars could not be completely eliminated. Additionally, microleakage and interfacial analysis may not reflect long-term clinical outcomes. Positive and negative controls and blank material controls were not included in the present study. Additionally, the potential influence of nitric acid digestion on different obturation materials cannot be completely excluded.

A significant methodological limitation of the present study is the unavailability of the original high-resolution SEM digital image files. Consequently, independent verifiability of the SEM gap measurements—including precise identification of the dentin–sealer–core interfaces, exact location of measured gaps, confirmation of calibrated scale bars, and complete verification of the representativeness of the shown images—is restricted. While image analysis and quantitative measurements were originally performed using integrated SEM software before data loss, the inability to re-examine raw high-resolution files requires that the micrographic interfacial findings be interpreted with caution and alongside the quantitative spectrophotometric dye extraction results.

## Conclusion

Within the limitations of this *in vitro* study, the BioRoot RCS/bioceramic-coated gutta-percha group (Group 1) demonstrated the lowest microleakage and the smallest mean interfacial gap among the experimental groups, whereas the BioRoot RCS/conventional gutta-percha group (Group 4) exhibited comparatively higher microleakage and larger gap values. No statistically significant difference was observed between the BioRoot RCS/ActiV GP (Group 2) and BioRoot RCS/EndoREZ (Group 3) groups. The spectrophotometric dye extraction method and SEM evaluation demonstrated comparable trends in assessing sealing performance under the conditions of this *in vitro* study. Further studies using larger sample sizes, complementary leakage assessment techniques, and long-term clinical evaluation are recommended to validate these findings.

## Data Availability

The datasets presented in this article are not readily available because it belongs to institution. Requests to access the datasets should be directed to dr.vinutham@jssuni.edu.in.
